# MEASURING BARRIERS TO LONG-TERM CARE RESIDENT PREFERENCES: DEVELOPMENT AND CONTENT VALIDITY TESTING

**DOI:** 10.1093/geroni/igac059.2858

**Published:** 2022-12-20

**Authors:** Laura Block, Ella Greenhalgh, Tonya Roberts

**Affiliations:** UW-Madison School of Nursing, Madison, Wisconsin, United States; UW-Madison School of Nursing, Madison, Wisconsin, United States; University of Wisconsin-Madison, Madison, Wisconsin, United States

## Abstract

Person-centered care (PCC), or delivery of care consistent with preferences, can improve outcomes for residents in long-term care (LTC). However, PCC has not been universally adopted. Few studies focus on measuring specific barriers to meeting resident preferences, hindering identification of actionable targets to improve PCC delivery. The purpose of this study was to develop and test the content validity of a survey that measures staff barriers to fulfilling resident preferences for daily care and activities. Item sets comprised of barriers and preferences were informed by a review of literature and a descriptive, qualitative study with 19 LTC staff. An expert panel (n=8) was convened to test content validity, and content validity indices (CVI) were calculated. The initial survey consisted of 12 item sets assessing whether knowledge gaps, resident characteristics (e.g. health needs), family, facility rules/policies, availability of options, or staffing/workload functioned as barriers to meeting resident preferences for daily care and activities (CVI = 0.76 barriers; CVI = 0.89 preferences). Revisions informed by expert feedback resulted in a total of 17 items sets. Second round testing with experts (n=10) revealed overall improved item sets (CVI = 0.77 barriers; CVI = 0.96 preferences). These findings indicate that the survey items have initial content validity in measuring staff barriers to meeting resident daily care and activity preferences. Further psychometric testing with LTC staff is needed prior to implementing the survey, which will represent the first tool of its kind for supporting measurement of staff-derived targets for PCC interventions and policy change.

